# Disinfection of Wounds and Instruments by a Solution of Formalin

**Published:** 1898-05

**Authors:** 


### FRENCH.

Under the Direction of Dr. Alexander Glass,
PHILADELPHIA.

Disinfection of Wounds and Instruments by a Solution of Formalin.—The most powerful sterilizing and deodorizing agent is the commercial solution of formalin. Formalin is twice as powerful as corrosive sublimate. This has been proven by taking a solution of 1 to 200, which was found to be ten times as strong as a solution of corrosive sublimate of 1 to 1000. As a deodorizing agent formalin possesses properties which are extraordinary. In tissues which have become gangrenous the odor is destroyed almost immediately when the parts have been dressed with a solution of 1 to 200; under various names formalin has been sold and proven to be a sterilizer and disinfectant of the first order. In solution of 1 to 200 it is very useful to sterilize instruments, and in this preparation it will not irritate the eyes or the skin when used. In wounds, gangrene, or the destruction of tissue this preparation is found to answer the purpose best, although some observers prefer to use a solution of 1 to 400; when mortification or destruction of tissue is present a solution of 1 to 1000 is not sufficient.—*Journ. de Pract.*

